# Antihypertensive and lipid lowering treatment in 70–74 year old individuals – predictors for treatment and blood-pressure control: a population based survey. The Hordaland Health Study (HUSK)

**DOI:** 10.1186/1471-2318-6-16

**Published:** 2006-10-19

**Authors:** Mette Brekke, Steinar Hunskaar, Jørund Straand

**Affiliations:** 1 Section for General Practice, Department of General Practice and Community Health, University of Oslo, Norway; 2 Section for General Practice, Department of Public Health and Primary Health Care, University of Bergen, Norway

## Abstract

**Background:**

In an elderly, community based population we aimed at investigating antihypertensive and lipid lowering medication use in relation to own and familiar cardiovascular morbidity and diabetes mellitus, as well as to lifestyle factors and general health. We also examined levels of blood pressure in untreated and treated residents, to investigate factors correlating with blood pressure control.

**Methods:**

A health survey carried out in 1997-9 in the county of Hordaland, Norway included a self-administered questionnaire mailed to 4 338 persons born in 1925-7. Drug use the day prior to filling in the questionnaire was reported. A health check-up was carried out, where their systolic and diastolic blood pressure (SBP and DBP), body mass index (BMI), and serum-cholesterol level were recorded.

**Results:**

One third of respondents used one or more antihypertensive drugs, while 13% of men and women were treated with a statin. Diabetes mellitus, own or relatives'cardiovascular disease, having quit smoking, physical inactivity, and overweight correlated with antihypertensive treatment. Mean blood pressure was lower in respondents not on treatment. Among those on treatment, 38% of men and 29% of women had reached a target BP-level of lower than 140/90 mm Hg. Own cardiovascular disease and a low BMI correlated with good BP-control.

**Conclusion:**

One third of 70–74 year old individuals living in the community used one or more antihypertensive drugs. Only around one third of those treated had reached a target BP-level of less than 140/90 mm Hg. Own cardiovascular disease and a low BMI correlated with good BP-control.

## Background

During the past decades, an increasing body of evidence has emerged on the importance of blood pressure as a risk factor for cardiovascular disease also in elderly people. Current European and US guidelines reinforce that doctors should not focus solely on hypertension, but include blood lipids and lifestyle factors in a multi-factorial risk assessment [1,2]. Since the first guideline on the management of hypertension were issued by the WHO in 1962 [3], subsequent versions have emerged at regular intervals. In general, the newer the guidelines, the lower the blood pressure (BP) cut-off points for intervention, and a corresponding trend also applies to the guidelines for blood lipids [4].

Most pharmacological interventions for modifying cardiovascular risk factors take place in general practice. However, two conflicting concerns have emerged. The first, often expressed by general practitioners (GPs), is that the cut-off points are now so low that only a minority of elderly persons will be regarded as "normal" or "healthy" without a need for drug treatment [5]. The second concern is that GPs' present treatment practice is insufficient, because hypertension and hyperlipidemia too often go unrecognized or are not treated aggressively enough [6,7].

The aim of this study was first, to analyze the level of elderly peoples' use of antihypertensive and lipid lowering drugs in relation to own and relatives' cardiovascular disease and diabetes mellitus, own lifestyle factors, BMI and general health status. The second aim was to investigate present BP in elderly people with or without treatment, and factors correlating with good BP-control.

## Methods

### The Hordaland Health Study (HUSK)

In 1997-9 a population based health survey was carried out in Hordaland County in western Norway (with capital city Bergen): The Hordaland Health Survey (HUSK) [8]. In total, 44 342 persons were invited, among whom 4 338 were 70–74 year old (born between 1925 and 1927) and living in Bergen and three nearby communities. The survey was carried out by the National Health Screening Service, Oslo (now The Norwegian Institute of Public Health) in cooperation with the University of Bergen.

### Questionnaire and measurements

A questionnaire was mailed to the invited persons, along with an invitation to a health check-up. The questionnaire included the following questions regarding drug consumption: Did you take any drugs yesterday? (yes/no). If yes: report the name of the drug, the reason for taking it, and whether you usually take this drug every day (yes/no). The questionnaire provided space for reporting up to nine different drugs, and participants were asked to continue on a separate sheet if needed. The questionnaire also addressed physical exercise, smoking, alcohol use, general health, and depressive symptoms, duration of formal education, previous or present diabetes mellitus and cardio- and cerebrovascular disease (including family history). Some variables were dichotomized during data analysis (see Table 1).

**Table 1:** Questions on lifestyle and health status

**Physical activity**
Which level of physical activity have you had during the last year?
• Easy exercise:*none, less than 1 hour/week, 1–2 hours/week, 3 hours or more/week*
• Hard exercise:*none, less than 1 hour/week, 1–2 hour/week, 3 hours or more/week*

**Use of alcohol**
• Are you totally abstinent from alcohol? *Yes/no*
• How many times a month do you usually drink alcohol? *Number of times:*
• How many glasses of beer, wine or spirits do you usually drink during a two-week period? *Number of glasses:*

**Smoking**
• Do you smoke cigarettes daily? *Yes/no*
• If you used to be a daily smoker, since when did you quit? *Number of years:*
• If you are or were a daily smoker, how many cigarettes do or did you usually smoke during a day? *Number of cigarettes:*
• For how many years have you been a daily smoker? *Number of years:*

**General health**
• How is your present general state of health? Bad/not quite good/good/very good*

**Depressed mood**
Have you felt down/depressed during the last two weeks? *Yes/a little/*
• considerably/very much**


* Less than good general health: bad or not so good

** Depressed mood: yes or a little

The completed questionnaires were gathered at the screening station. Here, systolic (SBP) and diastolic blood pressure (DBP), (average of the last two out of three recordings), as well as height and weight for calculating body mass index (BMI) were recorded. Blood samples were drawn for analyzing (among others) serum cholesterol.

The drugs reported to be used were subsequently coded according to the 1996- version of the Anatomical Therapeutic Chemical (ATC) classification system [9]. The ATC-groups included in this analysis were: C02 Anti-adrenergic agents, C03 Diuretics, C07 Beta blocking agents, C08 Calcium channel blockers, C09 Agents affecting the renin-angiotensin system, and C10 Lipid lowering agents.

Reported reasons for drug use were coded based on a modified list of diagnoses according to the International Classification for Primary Care (ICPC) [10].

### Ethics and statistics

The study was approved by the Regional Committee for Medical Research Ethics and the Norwegian Data Inspectorate.

Statistical analyses were performed using SPSS version 11. Bivariate comparisons were performed using chi-square test or independent samples t-test. Level of statistical significance was set at 5% (p < 0.05).

## Results

Out of the 4 438 elderly persons invited, 3 341 (77%) responded by filling in the questionnaire and attending the health check-up. They comprise the material for this study. 1 301 persons (39% of all; 585 men, 716 women) reported daily use of antihypertensives or statins. Mean number of these drugs per user was 1.62. Among the 445 statin users, 286 (64%) were also on antihypertensive treatment. Out of the 518 men (35%) and 626 women (33%) using antihypertensives, 51% reported "elevated blood pressure" as indication for use, 20% reported "cardiac problems," and 29% reported some other indications or gave no explicit reason for use.

The distribution of the antihypertensives according to ATC-groups was: anti-adrenergic agents: 95, diuretics: 233, beta blocking agents: 515, calcium channel blockers: 390, and agents affecting the renin-angiotensin system: 439.

Among persons who had suffered myocardial infarction, 70% of men and 82% of women were on antihypertensive drug treatment. For cerebral stroke, corresponding figures were 63% (men) and 47% (women), and for angina pectoris 67% (men) and 74% (women). Among men with diabetes mellitus, 58% were on antihypertensive treatment while this applied to 57% in women.

Cardiovascular morbidity in close relatives (parents, brothers or sisters) was strongly correlated with antihypertensive drug use (Table 2). Among persons with a positive family history of myocardial infarction, 42% of men and 38% of women were on treatment. For those with a family history of early (< 60 years of age) myocardial infarction, the numbers were 43% and 39%, for early (< 70 y) apoplexia 43% and 41%, respectively. Daily smoking was negatively correlated to antihypertensive treatment, as was doing physical exercise or having a BMI < 27 kg/m^2^. Formal education < 12 years, feeling depressed (women), and a less than good self-reported health all correlated positively to treatment.

**Table 2:** The Hordaland Health Study (HUSK). Own and familiar cardiovascular morbidity, lifestyle factors, and health status in 3 341 elderly persons (70 – 74 years old), using and not using antihypertensive drugs.

	Males (n = 1473)		Females (n = 1868)	
	
	*No drugs (n = 955 64.8%)*	*Drugs (n = 518 35.2%)*	*No drugs (n = 1260 66.8%)*	*Drugs (n = 626 33.2%)*
Myocardial infarction	63 (6.6)	149 (28.8)**	20 (1.6)	93 (14.9)**
Angina pectoris	68 (7.1)	138 (26.6)**	45 (3.6)	128 (20.4)**
Apoplexia	32 (3.4)	55 (10.6)**	32 (2.5)	28 (4.5)*
Diabetes mellitus	40 (4.2)	56 (10.8)**	52 (4.1)	65 (10.4)**
Family history of coronary disease	364 (38.1)	224 (43.2)**	566 (44.9)	346 (55.3)**
Family history of myocardial infarction < 60 y	129 (13.5)	96 (18.5)*	206 (16.3)	130 (20.8)*
Family history of apoplexia < 70 y	100 (10.5)	76 (14.7)*	173 (13.7)	118 (18.8)*
Daily smoking	158 (16.5)	57 (11.0)**	210 (16.7)	57 (9.1)**
Quit smoking	543 (56.9)	351 (67.8)**	283 (22.5)	170 (27.2)*
Alcohol > 14 times/month	79 (9.7)	48 (10.7)	45 (4.5)	13 (2.7)
No physical exercise	108 (12.0)	72 (14.5)	170 (15.3)	125 (22.1)**
Hard physical exercise	337 (38.8)	134 (28.5)**	213 (20.5)	88 (16.6)
BMI => 27 kg/m^2^	289 (30.3)	210 (40.5)**	431 (34.7)	314 (50.2)**
Education < 12 y	297 (57.4)	181 (65.1)*	573 (79.9)	304 (86.4)*
Depressed mood	29 (3.5)	11 (2.5)	37 (3.7)	32 (6.8)**
Less than good general health	212 (22.6)	180 (35.7)**	389 (31.9)	285 (46.5)**

Proportions, given in numbers and percentages, are compared using chi square tests.

*Significant difference between samples with versus without drugs (chi square test), p < 0.05.

**Significant difference between samples with versus without drugs (chi square t-test), p < 0.01

In the population on antihypertensive treatment, 23% reported angina pectoris, 21% myocardial infarction, 11% diabetes mellitus, and 7% apoplexia.

Statins were used by 195 men (13%) and 244 women (13%) (Table 3). Own and familiar cardiovascular disease, as well as diabetes mellitus correlated with statin use (bivariate analyses). Among those not using a statin, 84% of men and 92% of women had total cholesterol levels > 5 mmol/l, while corresponding figures for statin users were 56% of men and 80% of women.

**Table 3:** The Hordaland Health Study (HUSK). Own and familiar cardiovascular morbidity in 3 341 elderly persons (70 – 74 years old), using and not using lipid lowering drugs.

	Males (n = 1473)		Females (n = 1868)	
	
	*No drugs (n = 1278 86.8%)*	*Drugs (n = 195 13.2%)*	*No drugs (n = 1642 87.1%)*	*Drugs (n = 244 13.1%)*
Cardiac infarction	108 (8.4)	104 (53.3)**	52 (3.2)	61 (25.0)**
Angina pectoris	103 (8.1)	103 (52.8)**	104 (6.3)	69 (28.3)**
Apoplexia	72 (5.6)	15 (7.7)	46 (2.8)	14 (5.7)*
Diabetes mellitus	81 (6.3)	24 (12.3)**	92 (5.6)	25 (10.2)**
Family history of coronary disease	480 (37.6)	108 (55.4)**	749 (45.6)	163 (66.8)**
Family history of cardiac infarction < 60 y	179 (14.0)	46 (23.6)	264 (16.1)	72 (29.5)**
Family history of apoplexia < 70 y	149 (11.7)	27 (13.8)**	249 (15.2)	42 (17.2)

Proportions, given in numbers and percentages, are compared using chi square tests.

*Significant difference between samples with versus without drugs, p < 0.05

**Significant difference between samples with versus without drugs, p < 0.01

SBP was significantly higher in persons on antihypertensive treatment compared to persons not on BP-lowering medications (Table 4). DBP was higher for women in the treatment group, but not for men. Among those treated, 38% of men and 29% of women had reached a target level of BP < 140/90 (Table 4). Previous cardiovascular events, not being overweight, using a statin, and reporting a good general health (in women only) correlated with a successful BP-control in those on treatment (Table 5).

**Table 4:** The Hordaland Health Study (HUSK). Blood pressure (BP) in 3 341 elderly persons (70 – 74 years old), using and not using antihypertensive drugs.

	Males (n = 1473)		Females (n = 1868)	
	
	*No drugs (n = 955)*	*Drugs (n = 518)*	*No drugs (n = 1260)*	*Drugs (n = 626)*
*Systolic BP (mm Hg)*	144.5 (18.7) 91/210	147.5* (21.5) 88/223	145.6 (22.0) 98/229	153.4* (21.6) 84/220
Diastolic BP (mm Hg)	80.0 (10.7) 48/120	80.1 (12.1) 46/116	75.0 (12.6) 44/131	77.8* (12.1) 40/123
BP < 140/90 mmHg (%)	42.2	37.6	44.4	29.2**

Figures are given as means with standard deviation (SD) and range (minimum/maximum).

*Significant difference between samples with versus without drugs, independent samples t-test p < 0.01.

** Significant difference between sample with versus without drugs, chi square test p < 0.01

**Table 5:** The Hordaland Health Study (HUSK). Predictors for successful blood pressure-control in 1144 elderly persons (70–74 years old) on antihypertensive treatment.

	Males (n = 518)		Females (n = 626)	
	
	*BP < 140/90 n = 195*	*SBP ≥ 140 or DBP ≥ 90 n = 323*	*BP=<140/90 n = 183*	*SBP ≥ 140 or DBP ≥ 90 n = 443*
Cardiovascular disease	130 (66.7)	131 (40.6)**	73 (39.9)	118 (26.6)**
Diabetes mellitus	23 (12.3)	33 (10.4)	15 (8.5)	50 (11.5)
Family history of early CVD	50 (25.6)	99 (30.7)	62 (33.9)	150 (33.9)
Daily smoking	23 (11.9)	34 (10.8)	16 (9.1)	41 (10.0)
Quit smoking	133 (67.5)	218 (68.2)	50 (27.3)	120 (27.1)
Alcohol > 14 times/month	16 (9.4)	32 (11.5)	2 (1.4)	11 (3.2)
No physical exercise	29 (15.7)	43 (13.9)	39 (23.9)	86 (21.4)
Hard physical exercise	44 (25.0)	90 (30.6)	24 (15.4)	64 (17.3)
BMI => 27 kg/m^2^	63 (32.3)	147 (45.5)**	80 (43.7)	234 (52.8)*
Education < 12 years	68 (65.4)	113 (64.9)	81 (86.2)	223 (86.4)
Depressed mood	5 (3.1)	6 (2.1)	11 (8.1)	21 (6.3)
Less than good general health	76 (40.2)	104 (33.0)	94 (53.1)	191 (43.8)*
Statin treatment	60 (30.8)	68 (21.1)*	58 (31.7)	96 (21.7)*

Proportions, given in numbers and percentages, are compared using chi-square tests.

*p < 0.05

**p < 0.01

## Discussion

The high response rate (77%) contributes to strengthen the external validity of the findings. Furthermore, we consider data quality to be good, as drug names generally were correctly written and only few questionnaires had to be rejected because of inconsistencies.

However, some limitations have to be considered. First, people with a poor health, low socioeconomic status and an unhealthy lifestyle are generally known to be over-represented among non-respondents in health surveys [11], and unpublished HUSK-data reveal that in the elderly population, non-participants had lower education and income. We therefore find it likely that the 23% who did not participate in this survey use more drugs than the respondents. Second, the validity of elderly persons' self-reported drug use and reasons for use may be questioned. However, asking which drugs were taken *the previous day *(i.e. point prevalence) probably minimized recall bias [12]. Asking which drugs were actually taken also bypassed the problem of non-compliance, as we know that around 25% of prescribed drugs may not be used [13,14]. This also applies to BP- and lipid lowering drugs [15], which suggests that some of the non-users here probably had been prescribed some antihypertensive or lipid lowering drug.

We chose to include all use of drugs with ATC-codes C02, C03, C07, C08, or C09 in the analyses and to label them as "antihypertensive drugs", although some patients obviously used e.g. diuretics or beta-blockers for other cardiovascular reasons. Out of all users of the listed ATC-drugs, as many as 29% reported no explicit diagnostic reason for taking them, and some gave very unspecific or obviously wrong indications. In addition, as many as 63 persons reported "elevated BP" for use of drugs not relevant for antihypertensive treatment. Our figures here correspond well with results in a comprehensive British interview study among people aged 65 +, where 24% did not know the reason for taking their medications [13]. We therefore focused on our reliable registration of drug names, without paying attention to self-reported indications for use, which may represent a further limitation of this study.

In Western countries, elderly people are the major drug consumers and the average number of daily drugs used per person is increasing [13,16-19]. In another paper from this survey, we have reported that two thirds of men and three out of four women took at least one ATC-drug, with hypertension and other cardiovascular problems being by far the most common reasons for drug use [20]. In the elderly, cardiovascular drugs are generally reported to be the most commonly used drugs, though exceptions exist [13,16,18,21].

There were no significant differences between the two genders' total use of BP- and cholesterol lowering drugs, whereas a low formal education correlated with more use. This is also reported by others [16,22] and probably mirrors the well-known inverse relationship between social class and (risk of) cardiovascular disease [23]. In this survey, feeling depressed correlated strongly with total drug use [20], but logically this correlation was less pronounced for cardiovascular drugs, as compared to e.g. psychotropic drugs. Reporting a depressed mood only correlated positively with the use of antihypertensive or lipid lowering drugs in women.

Not surprisingly, performing no regular exercise or reporting a less than good general health were also associated with more use of BP- and cholesterol lowering drugs, corresponding to the results of others [24]. It may be surprising that being a non-smoker or having quit smoking both correlated positively with more drug use. That former smokers were those most likely to be on some medication, corresponds to what has been found in other studies [24,25]. One explanation may be that people who fall ill and are put on regular medication, tend to quit smoking, while smokers who remain healthy, more likely continue smoking. Other explanations are that a significant share of the heavy smokers probably has died before reaching an age of 70–74 years [26], and that smokers may be over-represented among the non-responders.

Use of alcohol every second day or more often did not correlate with antihypertensive treatment. Underreporting of actual drinking may have occurred, but contrary to smoking, regular, moderate alcohol consumption seems to be associated with less cardiovascular morbidity and mortality [27]. In an American study, no significant relationship was found between current use of alcohol and medications among persons 65 and over [24].

In the medical community, a tension exists between avoiding excessive use of medications for the elderly and to provide access to therapies that have beneficial effects on mortality, morbidity, functioning and quality of life [28,29]. This dilemma is especially pronounced for cardiovascular drugs, since they are so commonly used for primary and secondary risk prevention. Using drugs for secondary prevention in cardiovascular disease is effective in individuals 65 years and older, and prescription on this indication is recommended for all without contraindications [30]. However, drug treatment as primary prevention is becoming ever more present, even in older age groups, reflecting the new guidelines [1,2]. Implementing the 2003 European guidelines on CVD prevention [1] on a population from another Norwegian county, Getz et al estimated that around 90% of adults 50 years or older in fact were in need of some interventions due to their unfavorable cholesterol and/or BP values [5]. Because so few are regarded healthy according to these guidelines, the authors expressed serious concerns regarding medicalization, resource allocation, and sustainability within the healthcare system.

On the other hand: Our finding that less than 40% of those already on BP-lowering drugs had actually reached the recommended BP levels, indicates substantial potentials for improving their treatment. This finding, which is in line with others, indicate that a large proportion of treated patients still have inadequate control of their BP. In particular, being overweight correlated with a poor BP-control [7,31]. Corresponding to our findings, a recent Norwegian study showed that nearly two thirds of 75 year olds on antihypertensive treatment still had not reached a target BP-level [7].

## Conclusion

Around one third of 70–74 year old men and women living in the community used one or more antihypertensive drugs, and 13% of men and women used a statin. Diabetes mellitus, own or relatives' cardiovascular disease, being physically inactive, overweight, and being an ex-smoker correlated with antihypertensive treatment.

Among those on drug treatment, less than 40% of men and 30% of women had reached the recommended target values for blood pressure (below 140/90 mmHg), and being overweight correlated with poor BP control.

## Competing interests

The author(s) declare that they have no competing interests.

## Authors' contributions

JS and SH planned the study and carried out the data collection. MB carried out the analyses and drafted the paper. JS and SH participated in the discussion and in the writing of the article.

All authors read and approved the final manuscript.

## Pre-publication history

The pre-publication history for this paper can be accessed here:


